# Left Atrial Myxoma Presenting as Persistent Dizziness

**DOI:** 10.7759/cureus.26321

**Published:** 2022-06-25

**Authors:** Hayder Azeez, Zeinab A Abdulrahman, Tien Nguyen, Michael Tofano

**Affiliations:** 1 Internal Medicine, Mather Hospital/Zucker School of Medicine/Northwell Health, Port Jefferson, USA; 2 Cardiology, Mather Hospital/Zucker School of Medicine/Northwell Health, Port Jefferson, USA; 3 Medicine/Cardiology, Mather Hospital/Zucker School of Medicine/Northwell Health, Port Jefferson, USA

**Keywords:** emboli, echocardiogram, stroke, atrial myxoma, dizziness

## Abstract

Cardiac masses are divided into benign tumors and malignant tumors. The tumor can cause valvular obstruction and embolization phenomenon. To elucidate the etiology of cardiac masses, we rely on the use of echocardiograms in combination with the clinical picture of the patient. We describe an interesting case report of a 71-year-old woman who presented with persistent dizziness for one day. MRI brain showed multiple, small, scattered foci of acute infarction. The patient was treated with aspirin and atorvastatin. Transthoracic echocardiography showed a mass in the left atrium. Afterward, the tumor was removed surgically and histopathology was consistent with atrial myxoma.

## Introduction

Myxoma is the commonest type of cardiac masses existing in the left atrium presenting 80% of cases, whilst other cardiac masses are found in the right atrium [1]. Patients usually present with clinical features due to intracardiac obstruction or systemic embolization. More often than not, myxomas are found incidentally on cardiac imaging done for other purposes. Different imaging modalities, such as echocardiography, computed tomography, or magnetic resonance imaging, could be very helpful in identifying the nature of the mass in symptomatic patients [2-3].

## Case presentation

Our patient is a 71-year-old woman with a medical history of hypertension, hyperlipidemia, and right parietal craniotomy with tumor resection (no available data regarding the tumor type) who presented to the emergency department with persistent dizziness for one day. The patient described the dizziness as a spinning sensation associated with nausea, mild headache, blurry vision, and difficulty walking. The patient denied any history of a similar attack, falls or balance disorders, recent illness, fever, audiological symptoms, confusion, weakness, numbness, shortness of breath, chest pain, palpitations, or change in speech.

**Figure 1:**
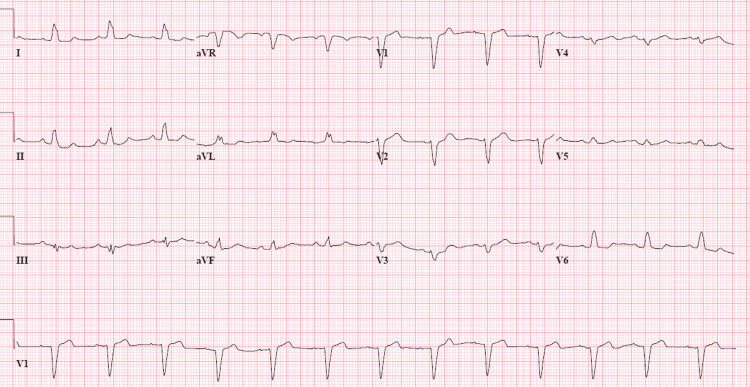
EKG on admission showed sinus rhythm and LBBB LBBB: left bundle branch block

On physical examination, blood pressure was 137/70 mmHg and heart rate was 79 beats per minute in the supine position while the blood pressure was 123/58 mmHg and the heart rate was 77 beats per minute in the standing position. There were no signs of emboli on the skin or nails. She did not show any obvious nystagmus or focal neurological sign. Examination of the heart and lungs showed clear lung bilaterally, normal heart sounds, and regular rhythm. There was no murmur appreciated by auscultation. There was no occurrence or change in dyspnea or murmur on changing posture. There was no deafness, gait disorder, or cerebellar signs. No previous EKG was found in the medical record to compare with EKG in admission. EKG on admission showed sinus rhythm and left bundle branch block (LBBB) (Figure 1).

**Figure 2:**
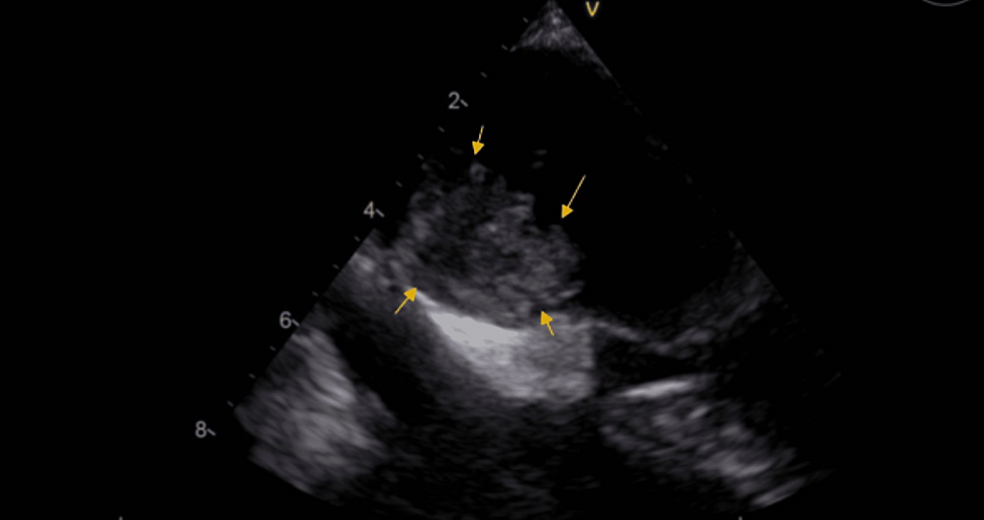
Transthoracic echocardiography showing a 2 cm X 2.1 cm mobile mass in the left atrium

**Figure 3:**
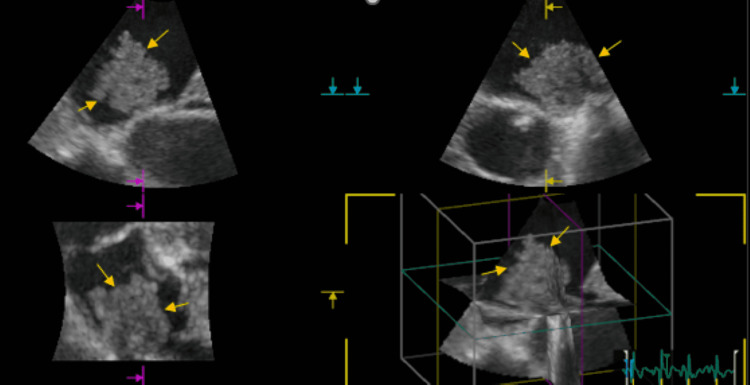
3D echocardiography showing a 2 cm X 2.1 cm mobile mass seen in the left atrium

Doppler of lower extremities was negative for deep venous thrombosis. CT scan of the head showed postoperative changes consistent with right parietal craniotomy, encephalomalacia, and gliosis within the right posterior frontal lobe representing sequelae of prior surgery. Computed tomography angiography with contrast of the head and neck arteries didn't show any evidence of an aneurysm, vascular malformation, occlusion, dissection, or stenosis. MRI of the brain showed multiple, small, scattered foci of acute infarction that were seen throughout the brain parenchyma, both supratentorial and infratentorial. The pattern of disease suggests emboli as a possible etiology. The patient was admitted to the stroke unit to evaluate the etiology of her stroke. The patient did not receive a tissue plasminogen activator as she presented to the emergency department out of the treatment window. The patient was treated with aspirin 81 mg and atorvastatin 40 mg. One day later, transthoracic echocardiography showed an ejection fraction of 55%, and a mobile mass was seen in the left atrium measuring about 2 cm X 2.1 cm, connected to the inter-atrial septum and suggestive of myxoma (Figures 2-3). 

**Figure 4:**
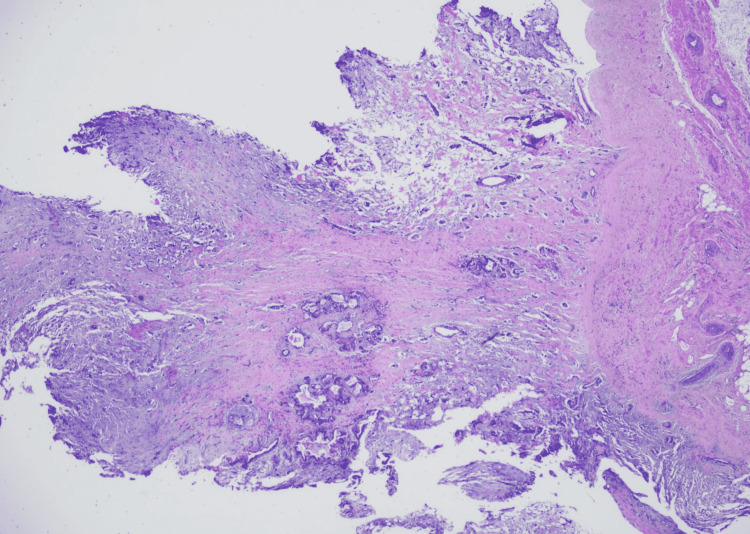
Low cellularity lesion with a myxoid background, stellate spindled cells, and well-differentiated mucinous glands, which is consistent with atrial myxoma

Afterward, the tumor was removed surgically and histopathology was consistent with atrial myxoma (Figure 4).

## Discussion

Patients with a myxoma may present with symptoms referable to intracardiac obstruction (dyspnea, syncope), symptoms related to systemic embolization, or constitutional symptoms related to interleukin production. Patients with myxoma infrequently present with dizziness [4].

The anatomic location of the tumor determines cardiovascular symptoms and clinical examination findings [5]. Patients with left atrial myxomas may develop mitral valve regurgitation or stenosis depending upon the site of attachment [6]. When the tumor is located close to the mitral valve orifice, it results in obstruction and presents with symptoms of left heart failure symptoms and pulmonary congestion. Local invasion of the tumor can lead to conduction abnormalities and arrhythmias [7]. The most common presentation of atrial myxomas is dyspnea with exertion followed by orthopnea, paroxysmal nocturnal dyspnea, and pulmonary edema [8]. Careful physical examination, including good cardiac auscultation, plays a vital role in the initial diagnosis. Similar to the left side, the obstructive symptoms of right atrial myxomas depend on the site of attachment. The myxoma can be at the level of the tricuspid valve, causing obstructive symptoms that mimic tricuspid stenosis. Rarely, these patients can present with tricuspid regurgitation. A murmur may be appreciated over the tricuspid region. Over time, symptoms of right-sided heart failure will develop such as pedal edema, hepatic congestion, exertional dyspnea, and ascites. Massive obstruction can lead to syncope and decompensated right-sided heart failure [9]. Similar to the left side, right-sided myxomas can also invade the myocardium causing conduction abnormalities and associated arrhythmias.

Evidence of systemic embolization was present in 29% of patients, and 20% had neurologic deficits [10]. With myxomas, the incidence of embolization is associated with smaller size (≤4.5 cm) and softer tumors [10]. Owing to the high systolic left ventricular pressure, embolization due to left atrial myxomas commonly affects the central nervous system and retinal arteries. Embolization can also occur in the lower extremities (commonly, the iliac and femoropopliteal), viscera, spleen, adrenals, kidneys, and even the abdominal aorta [10]. The patient can present with dyspnea, transient ischemic attack, hemiplegia, syncope, loss of vision, arrhythmia, and chest pain. Symptoms may include Raynaud’s phenomenon, spotty skin pigmentation, abdominal pain, diarrhea, and other peripheral signs of embolization [2]. Embolization in right atrial myxomas can be of two types: (1) pulmonary artery embolism or systemic embolization if the patient has a patent foramen ovale or an atrial septal defect [11]; (2) pulmonary artery embolization is often life-threatening and may require an immediate pulmonary embolectomy to prevent the development of pulmonary artery hypertension and consequent right ventricular dysfunction [12]. Given its accessibility, echocardiography remains the key in the evaluation of a suspected cardiac mass, whereas CT and MRI would be helpful in identifying the type of tumor and the size and location of the cardiac mass. The imaging modalities will determine eventual management [13-14].

As the possibility of systemic embolization and cardiovascular complications, including sudden death are high, resection of the tumor is recommended following standard preoperative evaluations [15-16]. The outcomes of surgical resection are commonly very good, with most series reporting an operative mortality rate under 5% [14-15].

The post-surgical course is generally smooth. However, complications such as atrial arrhythmias or atrioventricular conduction abnormalities were reported postoperatively in 26% of patients in one series [15]. Recurrence is more common in patients whose primary tumor was multicentric [10]. For recurrent atrial myxoma, cardiac autotransplantation (with atrial reconstruction) or transplantation are considerable options [17-18].

## Conclusions

A cardiac neoplasm may be found incidentally during clinical assessment or it could present with symptoms. Cardiac myxomas might be the source of systemic thromboembolism and stroke. In our case, we highlight the importance of considering cardiac myxomas within the differential diagnosis for a healthy patient presenting with persistent dizziness. Also, we suggest keeping the possibility of systemic embolization and stroke in mind if the myxoma is small (≤4.5 cm).
